# Transgenic mice over-expressing carbonic anhydrase I showed aggravated joint inflammation and tissue destruction

**DOI:** 10.1186/1471-2474-13-256

**Published:** 2012-12-20

**Authors:** Yabing Zheng, Lin Wang, Wei Zhang, Hengwei Xu, Xiaotian Chang

**Affiliations:** 1 Medical Research Center of Shandong Provincial Qianfoshan Hospital, Shandong University, Jingshi road 16766, Jinan, Shandong 250014, P. R. China; 2 Research Center for Medical Biotechnology, Shandong Academy of Medical Sciences, Jingshi road 18877, Jinan, Shandong 250062, P. R. China; 3 The Secondary People’s Hospital of Jinan, Jingyi road 148, Jinan, Shandong, 250001, P. R. China; 4 Department of Pharmacy, Shandong Provincial Tumor Hospital, Jiyan road 440, Jinan, Shandong 250117, P. R. China

**Keywords:** Carbonic anhydrase I (CA1), New bone formation, Transgenic mice, collagen-induced arthritis (CIA), Ankylosing spondylitis (AS), Rheumatoid arthritis (RA)

## Abstract

**Background:**

Studies have demonstrated that carbonic anhydrase I (CA1) stimulates calcium salt precipitation and cell calcification, which is an essential step in new bone formation. Our study had reported that CA1 encoding gene has a strong association with rheumatoid arthritis (RA) and ankylosing spondylitis (AS), two rheumatic diseases with abnormal new bone formation and bone resorption in joints. This study investigated the effect of CA1 on joint inflammation and tissue destruction in transgenic mice that over-express CA1 (CA1-Tg).

**Methods:**

CA1-Tg was generated with C57BL/6J mice by conventional methods. CA1-Tg was treated with collagen-II to induce arthritis (CIA). Wild-type mice, CA1-Tg treated with bovine serum albumin (BSA) and transgenic mice over-expressing PADI4 (PADI4-Tg), a gene known to be involved in rheumatoid arthritis, were used as controls. Histochemistry and X-ray radiographic assay were used to examine joint destruction. Western blotting and real time-PCR were used to examine CA1 expression.

**Results:**

CIA was observed in 60% of CA1-Tg, 20% of PADI4-Tg and 20% of wild-type mice after collagen injections. No CIA was found in CA1-Tg mice that received injections of BSA. The arthritic score was 5.5 ± 0.84 in the CA1-Tgs but the score was less than 2 in the injected wild-type mice and the PADI4-Tgs. The thickness of the hind paws in the CA1-Tgs was 3.46 ± 0.11 mm, which was thicker than that of PADI4-Tgs (2.23 ± 0.08 mm), wild-type mice (2.08 ± 0.06 mm) and BSA-treated CA1-Tgs (2.04 ± 0.07 mm). Histochemistry showed obvious inflammation, synovial hyperplasia and bone destruction in the joints of CA1-Tg that was not detected in PADI4-Tgs or wild-type mice. X-ray assays showed bone fusion in the paws and spines of CA1-Tg mice.

**Conclusion:**

Over-expression of CA1 may aggravate joint inflammation and tissue destruction in the transgenic mice.

## Background

Abnormal new bone formation and bone resorption are the most distinctive features of ankylosing spondylitis (AS) [1-3]. Histopathological experiments have demonstrated that severe forms of AS correlate significantly with villous chronic synovitis, including obliterating vascularitis, fibrosclerosis, necrosis and calcification of disintegrated synovial structures [4]. Using a proteomic approach, we previously screened novel AS-specific proteins by simultaneously comparing the expression profiles of synovial membranes from patients with AS, rheumatoid arthritis (RA) and osteoarthritis (OA). That proteomic study revealed significantly increased expression of carbonic anhydrase I (CA1) in the synovial membrane of AS patients compared with those of RA and OA patients. Immunohistochemistry and western blotting analyses confirmed the above findings [5]. Furthermore, we also reported that methazolamide, an anti-CA drug, may improve AS symptoms by a case report [6]. CA1 is a member of the carbonic anhydrase (CA) family, and it catalyzes the reversible hydration and dehydration reactions of CO_2_/H_2_CO_3_[7]. In vitro assays have demonstrated that CA1 not only enhances the hydration reaction but also promotes the formation of CaCO_3_[8,9]. Calcium salt precipitation is an important step in bone formation. Thus, the increased CA1 expression in the synovium of AS patients may lead to improper mineralization by accelerating calcium salt deposition [5]. We recently found that an increased expression of CA1 stimulates calcium precipitation and ossification in cultured Saos-2 cells, a human osteosarcoma cell line [10]. We also found significant association between the CA1 DNA polymorphism and susceptibility to AS risk that the gene encoding CA1 is susceptible to AS and RA risk. However, no study has demonstrated a role for CA1 over-expression in the processes of new bone formation and bone resorption of joint tissue in physiological condition.

In this study, we generated transgenic mice that over-expressed CA1 (CA1-Tg) and injected the animals with collagen II (cII) to induce arthritis (CIA). We examined the clinical score, arthritic incidence and footpad swelling in CA1-Tg mice, and we used wild-type mice and transgenic mice that over-express PADI4 (PADI4-Tg), a gene known to be involved in RA [11], as controls. X-ray assays and histochemistry were employed to examine joint destruction in these mice. The purpose of this study was to use transgenic mice to determine the effect of CA1 over-expression on joint inflammation and tissue destruction.

## Methods

### Generation of transgenic mice that over-expressed CA1

Mouse full-length CA1 cDNA was amplified by reverse transcription PCR (RT-PCR) using a kit from Invitrogen (USA) and confirmed by sequencing analysis. The CA1 cDNA was inserted downstream of the CMV promoter in the pCDNA3.1 (+) vector between the BamHI and XhoI cloning sites. The completed plasmid was linearized by digestion with endonuclease BglII. The entire transgenic expression cassette including the CMV promoter, the CA1 full-length cDNA and the BGH polyA signal was not disrupted. The DNA fragments were gel-purified, dissolved in Tris–HCl–EDTA at a final concentration of 5 ng/μl and injected into the pronuclei of fertilized zygotes harvested from C57BL/6J mice using conventional methods to generate the transgenic mice [12]. This experiment was completed in Dr. Zhang Lianfeng’s laboratory at the Institute of Laboratory Animal Science at the Chinese Academy of Medical Sciences.

The transgenic founder (F0) mice were mated with wild-type C57BL/6J mice to produce the F1 generation, and CA1 expression was detected in the F1 transgenic mice. The F1 transgenic lines were mated with wild-type C57BL/6J mice to produce F2 mice, which were used for phenotypic analyses. The wild-type mice were also obtained from Institute of Laboratory Animal Science at the Chinese Academy of Medical Sciences. The transgenic mice that over-expressed PADI4 were generated in the same manner.

Mouse genomic DNA was extracted from tail biopsies. PCR was performed in a 20 μl reaction volume containing 1 μg of genomic DNA and the primers used for cloning the CA1 gene. The sense primer was 5^′^-GCAAGCTTCATGAGACCTAGAATGAAGTAT-3^′^, and the antisense primer was 5^′^-CGGGATCCTTTACTGCATTAGATTTTCGAG-3^′^. These primers amplify a 374 bp sequence from the pCMV promoter. The PCR product was examined using electrophoresis on 1% agarose gel.

Synovial tissues were dissected from CA1-Tg (n = 5), PADI4-Tg (n = 6) and wild-type (n = 6) mice. CA1 mRNA expression was quantified using real-time PCR. The thymus, spleen, liver, lungs and kidneys were dissected from CA1-Tg (n = 5), PADI4-Tg (n = 6) and wild-type mice. The levels of CA1 protein in these organs were examined using western blotting.

The procedures involving animals were approved by the Institutional Animal Care and Use Committee of the Chinese Institute of Laboratory Animal Science (GC-08-2018), and the mice were housed in an Association for Assessment and Accreditation of Laboratory Animal Care (AAALAC)-accredited facility. The Ethics Committee of Shandong Provincial Qianfoshan Hospital approved the study and the protocol.

### Collagen-induced arthritis

Bovine collagen type II (cII) (400 μg, Sigma, USA) was injected into the tails of CA1-Tg (n = 10), PADI4-Tg (n = 10) and wild-type (n = 10) mice (8 weeks old, male). CA1-Tg (n = 10) were also treated with bovine serum albumin (BSA) as controls. cII was dissolved overnight at 4°C in 10 mM acetic acid at a concentration 4 mg/ml and emulsified in an equal volume of complete Freund’s adjuvant (CFA, Sigma, USA) containing 100 mg heat-killed (strain H37Ra, Sigma, USA). These mice were injected with the same dose of cII in incomplete Freund’s adjuvant (IFA, Sigma) 3 weeks later. The severity of CIA was determined by monitoring the incidence of CIA, the clinical score and the thickness of the hind paws. The incidence was expressed as the percentage of mice that showed visible symptoms of arthritis. Clinical scoring was performed using an established scoring system as described in Von Delwig’s study [13]. The score for each paw was based on the degree of swelling and periarticular erythema using a scale of 0–3 as follows: 0 = no evidence of erythema or swelling, 1 = erythema confined to one joint region only, 2 = erythema and swelling limited to one joint region only and 3 = severe erythema and swelling extending from the ankle to the midfoot (tarsal) joint and involving both joint regions. Scores from all four paws were added to provide the total score for each mouse. The maximum possible score per mouse was 12. The thickness of the hind paws was measured using vernier calipers (reading error of 0.1 mm, Mitutoyo, JAP). Control-treated CA1-Tg (n = 10) mice were administered with BSA.

The development of disease was monitored for the period indicated. At 120 days after the first injection with cII, the mice were sacrified, and their paws were dissected, fixed in 10% formaldehyde (pH 7.4) and then embedded in paraffin wax. Serial sections (5 mm) of the joints were prepared and then stained using hematoxylin to evaluate pannus formation, synovial cell infiltrate and cartilage/bone erosion.

The data are representative of three independent experiments. The clinical scores and paw thickness were analyzed using a two-way analysis of variance. The incidence of CIA was analyzed using Fisher’s exact test.

### X-ray radiographic assessment

CA1-Tg mice, wild-type mice, PADI4-Tg mice and CA1-Tg mice treated with BSA were evaluated at 120 days after the first injection of CII. The mice were anesthetized and irradiated using a digital mammography X-ray as described by Zähringer [14]. Digital images of the vertebrae and joints were obtained using the following exposure parameters: mammography film, 23 kV and 32 mAs; Inc, -2; AGD, 0.52 mGy; ESE, 1.36 mGy. This experiment was completed in the Radiological Department of the Shandong Tumor Hospital.

### Real-time PCR

Total RNA was extracted from animal tissues using a total RNA kit (Omega, USA) according to the manufacturer’s protocol. The concentrations of total RNA were determined using a spectrophotometer. The cDNA was prepared with 500 ng of total RNA from each sample using oligo-dT primers and the PrimeScriptTM RT-PCR Kit (TaKaRa, Japan). Quantitative PCR analysis was performed using a LightCycler 480 thermocycler (Roche). Taqman real-time PCR was conducted in 10 μl reaction mixtures containing 5 μl of SYBR Green (Toyobo, Japan), 1 μl of each primer, 1 μl of cDNA and 2 μl of H_2_O. The optimized thermal cycling program was as follows: step 1, 95°C for 180 s; step 2, 95°C for 10 s, 59°C for 10 s and 72°C for 25 s (45 cycles); and step 3, a melting curve analysis over 59–95°C in 0.5°C increments. The specificity of the individual amplification reactions was assessed by examining the melting curves to confirm the presence of a single gene-specific peak with the characteristic melting temperature of the expected product. The primers for CA1 are as follows: 5^′^-GCTACAGGCTCTTTCAGTT-3^′ ^and 5^′^-GACTCCATCCACTGTATGTT-3^′^. The PCR results were normalized to the level of GAPDH expression. Two reactions were performed simultaneously for each sample. One reaction was performed to determine the expression of mRNA of the target gene, and the second reaction was performed to determine the level of GAPDH. The experiment was performed in triplicate, and the PCR products were confirmed using a melting-curve analysis. The relative expression of mRNA was calculated using the comparative threshold cycle (Ct) method according to the following formula: Ratio =2^–ΔΔCt^ = 2^-ΔCt(sample)-ΔCt(control) ^where ΔCt = Ct of the target genes – Ct of the endogenous control gene (GAPDH). The relative target gene expression was calculated by normalizing to the expression of GAPDH mRNA. The copy number of the target mRNA in each sample was calculated automatically using data analysis software. The levels of CA1 are expressed as the median and range. Significant differences were assessed using the Mann–Whitney U-test where p < 0.05 was considered statistically significant.

### Western blotting

Animal tissues were homogenized in RIPA buffer (Beyotime, China) supplemented with protease and phosphatase inhibitors on ice and centrifuged at 16,000x g for 5 min at 4°C. The supernatant was collected, and the protein concentration was determined using the BCA Protein Assay Kit (Beyotime, China). Thirty micrograms of total protein was separated on a 12% SDS-polyacrylamide gel and transferred onto a PVDF membrane. The membrane was rinsed with wash solution and incubated overnight at 4°C with anti-human carbonic anhydrase I antibody (Santa Cruz, USA) at a dilution of 1:500. The antibody was prepared by immunizing a mouse with a peptide comprising carbonic anhydrase I amino acids 33–80, which are near to the N-terminus. The manufacturer confirmed no cross-reactivity with other carbonic anhydrases. Immunosignals were visualized using the Protein Detector BCIP/NBT Western Blotting Kit (Beyotime, China) following the manufacturer’s instructions. The quantification was conducted using ImageQuant 5.2 software. To normalize the sample loading, a separate membrane was prepared in the same manner and was probed using an anti-GAPDH antibody (Santa Cruz, USA).

### Statistical analyses

All data are presented as the mean ± SD. Comparisons between two groups were performed using unpaired Student’s t-tests. Multiple intergroup comparisons were performed using one-way ANOVAs. The incidence of mice that developed the disease was analyzed using Fisher’s exact test. We considered p values <0.05 to be significant.

## Results

### Generation of CA1-Tg mice

To determine the potential role of CA1 in arthritis and AS, C57BL/6J transgenic mice that over-expressed CA1 were generated. Five transgenic founders (#1, #2, #4, #33, and #37) were generated and identified based on a PCR analysis of their genomic DNA. The transgenic founder (F0) mice were mated with wild-type C57BL/6J mice to produce the F1 generation, and the CMV/CA1 gene was identified based on a PCR analysis of their genomic DNA (data not shown). These founder mice did not show significant differences of phenotype, growth rate, body weight and fertility among them. All of five founders were used to generate F2.

### CA1 expression in the transgenic mice

**Figure 1:**
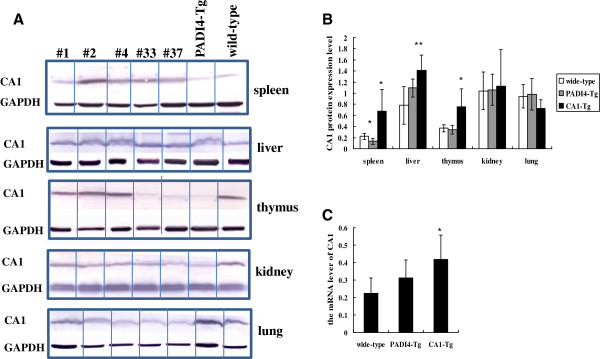
**The expression of CA1 in various tissues of CA1-Tg mice.** (**A**) The CA1 expression was examined in the spleen, liver, thymus, kidneys and lungs of CA1-Tg mice (n = 5), PADI4-Tg mice (n = 6) and wild-type mice (n = 6) using western blotting. (**B**) The expression levels of CA1 were semi-quantified by normalizing the expression of GAPDH in the protein extracts. C. The CA1 mRNA expression level was quantified in synovial tissues of CA1-Tg mice, PADI4-Tg mice and wild-type mice using real-time PCR. * p < 0.05 and ** p < 0.01.

CA1 protein levels were determined in the lung, kidney, spleen, thymus and liver of these transgenic mice (n = 5, 8 weeks old) using western blotting. CA1 protein (29 kDa) levels were 3.05-fold (p = 0.02) higher in the spleen, 1.8-fold (p = 0.005) higher in the liver, and 2.04-fold (p = 0.016) higher in the thymus of CA1-Tg mice compared with wild-type mice (n = 6, 8 weeks old). The expression of CA1 was unchanged, low or was not detected in other organs of the CA1-Tg mice, such as the lungs (p = 0.07) and kidneys (p = 0.767), compared with controls. The levels of CA1 protein in the various organs of the PADI4-Tg mice (n = 6, 8 weeks old) were similar to those of the wild-type mice. These results are shown in Figure 1A and B.

The level of CA1 mRNA in the synovial tissues of these F1 mice (n = 5) was quantified using real-time PCR. Compared with the expression of CA1 in synovial samples from wild-type mice (n = 6) and PADI4-Tg mice (n = 6), CA1 mRNA was increased 1.85-fold (p = 0.016) in the tissues of CA1-Tg mice. There was no significant difference in CA1 mRNA level of the synovial membranes between PADI4-Tg mice and wild-type mice (p = 0.145). These results are shown in Figure 1C.

### Collagen-induced arthritis in transgenic mice over-expressing CA1

**Figure 2:**
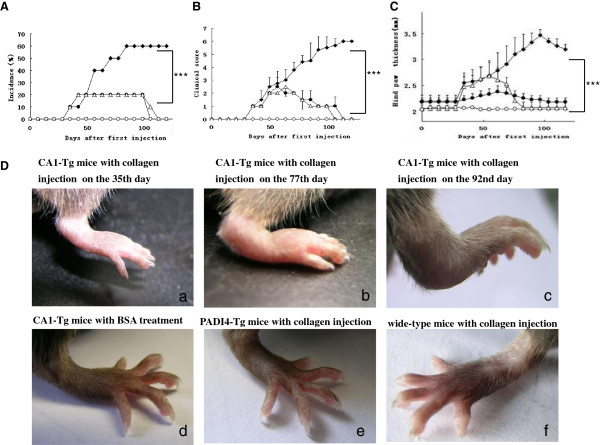
**Clinical symptoms of CIA in collagen-injected CA1-Tg mice.** The incidence of arthritis (**A**), clinical scores (**B**) and hind-paw thicknesses (**C**) were measured in CA1-Tg mice (filled squares, n = 10), PADI4-Tg mice (filled circles, n = 10) and wild-type (empty triangle, n = 10) mice at different time points after the first injection of collagen. CA1-Tg mice that received BSA were also included as a control (empty circles, n = 10). (**D**) Evident CIA appeared in the hind paws of CA1-Tg mice. **a** The collagen-injected CA1-Tg mice on the 35th day after the first injection of collagen, **b** collagen-injected CA1-Tg mice on the 77th day, **c** collagen-injected CA1-Tg mice on the 92nd day, **d** BSA-treated CA1-Tg mice on the 77th day, **e** collagen-injected PADI4-Tg mice on the 77th day, and **f** collagen-injected wild-type mice on the 77th day.

The arthritic incidence and the CIA clinical scores of CA1-Tg mice were compared with those of PADI4-Tg mice and wild-type mice. CA1-Tg mice that were injected with BSA were also used as controls. The incidence of CIA was 60% (6/10) in CA1-Tg mice, 20% (2/10) in PADI4-Tg mice, 20% (2/10) in wild-type mice and 0% (0/10) in CA1-Tg mice injected with BSA on the 84th day after the first injection (p = 0.017) (Figure 2A).

The arthritis severity score was 5.5 ± 0.84 for the CA1-Tg mice on the 98th day after the first injection, but the score was less than 2 for the wild-type mice and the PADI4-Tg mice under the same conditions. No clinical signs of arthritis were found in the CA1-Tg mice treated with BSA (p = 0.00001, Figure 2B).

The hind-paw thickness of the CA1-Tg mice was 3.46 ± 0.11 mm, which was thicker than that of PADI4-Tg mice (2.23 ± 0.08 mm), wild-type mice (2.08 ± 0.06 mm) and BSA-treated CA1-Tg mice (2.04 ± 0.07 mm; p = 0.00001, Figure 2C). The hind paws of the CA1-Tg mice showed significant edema, redness and ankylosis with pronounced loss of function on the 92nd day after the first injection of collagen, whereas the hind paws of all immunized PADI4-Tg mice and wild-type mice appeared normal (Figure 2D).

**Figure 3:**
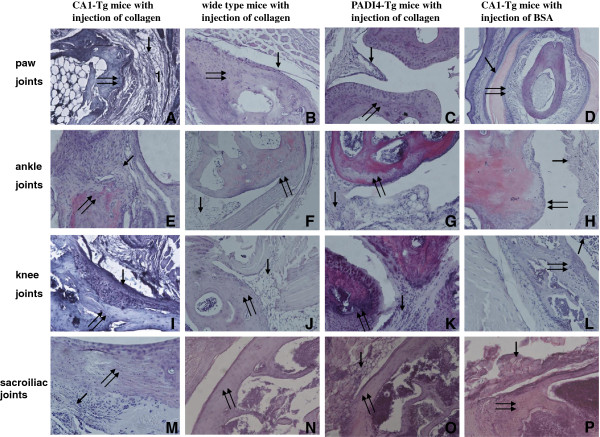
**Histochemical examination of collagen-induced arthritis in CA1-Tg mice.** Tissue structures of the paw (**A-D**), knee (**E-H**), ankle (**I-L**) and sacroiliac joints (**M-P**) were examined using histochemistry. Synovial hyperplasia and inflammation, cartilage destruction and bone resorption with pannus formation were detected in the arthritic joints of CA1-Tg mice (n = 5). The single arrow indicates the synovial membrane, and the double arrow indicates the joint cartilage. Magnification: ×400.

Five mice were selected randomly from the collagen-injected CA1-Tg mice with CIA (n = 5), the PADI4-Tg mice (n = 5), the wild-type mice (n = 5) without CIA and the BSA-treated CA1-Tg mice (n = 5) to examine joint tissue structure. Histological examination detected evident cell hyperplasia and diffused lymphoid cell infiltrate; fibrin deposition; cartilage-surface erosion; and bone destruction in the joints of the paw, ankle, knee and sacroilium of all five CA1-Tg mice with CIA. The joints of the PADI4-Tg mice and the wild-type mice that were injected with collagen and the CA1-Tg that were injected with BSA exhibited a smooth articular surface. No significant synovial inflammation or cartilage surface erosion was observed in these controls. These results are shown in Figure 3.

**Figure 4:**
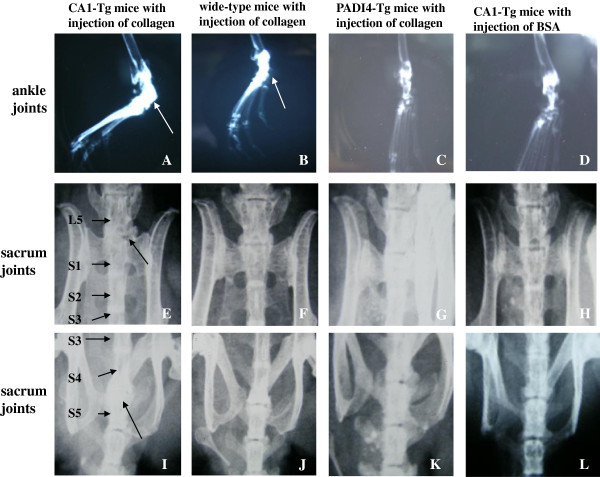
**X-ray images of joint tissue destruction in the ankle- and lumbar-spine vertebrae of collagen-injected CA1-Tg mice.** Evident bone formation (indicated with an arrow) was observed in the hind paws of immunized CA1-Tg mice (n = 5) (**A**) but not in the paws of wild-type mice (n = 5) (**B**), PADI4-Tg mice (n = 5) (**C**) or BSA-treated CA1-Tg mice (n = 5) (**D**). The disk space disappeared, and a chondrophyte/osteophyte was fused with the neighboring vertebral bodies between L5 and S1 (**E**) as well as S4 and S5 (**I**). There was no apparent bone fusion in the lumbar spine vertebrae of collagen-injected wild-type mice (**F, J**), PADI4-Tg mice (**G, K**) or BSA-treated CA1-Tg mice (**H, L**).

The destruction of the joint tissue described above in the collagen-treated CA1-Tg mice (n = 5), collagen-treated PADI4-Tg mice without CIA (n = 5), collagen-treated wild-type mice (n = 5) and BSA-treated CA1-Tg mice (n = 5) was examined using digital mammography X-ray imaging. In collagen-treated CA1-Tg mice, the contours of the cartilaginous tissues were diffuse due to chondrophyte formation. Progression of spondylitis was observed in each CA1-Tg animal that developed CIA. In the three CA1-Tg mice treated with collagen, the disk space disappeared, and chondrophyte/osteophyte fusion with the neighboring vertebral bodies was observed that replaced the original intervertebral disk. In the collagen-treated PADI4-Tg mice, the wild-type mice and the BSA-treated CA1-Tg mice, the growth plates were normal, and the end plates of the vertebral bodies appeared as sharp, solid lines. In addition, the hind paws of the injected CA1-Tg mice treated with CIA exhibited bone formation in the ankle joints. In contrast, the hind paws of the collagen-immunized PADI4-Tg mice and wild-type mice as well as the BSA-treated CA1-Tg mice appeared similar to those of wild-type mice that had not received any treatment and displayed no apparent bone formation. These X-ray results are shown Figure 4.

The above experiment was repeated for 3 times and we got similar results. The data are representative of three independent experiments. All F2 from different founders were used for the experiment. There was no significant difference among these F2.

## Discussion

In our study, transgenic mice that over-expressed CA1 were markedly susceptible to collagen treatment and showed a high incidence and serious symptoms of arthritis. X-ray images revealed that the disk space in the spinal regions had disappeared, that neighboring vertebral bodies had fused and that the bone density had increased in the paws and spines of collagen-immunized CA1-Tg mice. In contrast to CA1-Tg mice, clinical and histological evaluation showed a normal progression of disease parameters in the collagen-immunized wild-type mice and PADI4-Tg mice. The growth plates were normal in PADI4-Tg and wild-type mice. Histological examination also revealed obvious cartilage loss, synovial inflammation and bone destruction in the paw, ankle, knee and sacroiliac joints of CA1-Tg mice with CIA but not in the PADI4-Tg and wild-type mice. Thus, our study suggests that over-expression of CA1 may accelerate the joint inflammation and tissue destruction of the mice.

The inflammation, bone erosion and ossification of sacroiliac joints and the spine are histopathological features of AS [15]. Mark et al. established transgenic mice that expressed human HLA-B27. Using radiographic and histological techniques, they detected severe axial skeletal kyphosis and ankylosis accompanying an inflammatory and fibrotic process at the end plates and enthesis [16]. Their observations were similar to the observations of our animal model. These animals developed a spontaneous multisystem disorder that resembles the spondylitis observed in AS. Although the histological features of the synovial membranes of the peripheral joints in AS are similar to those of RA, fibroblastic proliferation, intimal cell hypertrophy, diffuse lymphocyte and histiocyte infiltration and the presence of fibrin were less marked in the joint tissues of AS than in joint tissues of definite RA. Decreased intimal cell hypertrophy and fibroblastic proliferation were observed in synovial tissues of AS compared with RA [17]. In the synovial tissue of CA1-Tg mice with CIA, intimal cell hypertrophy and fibroblastic proliferation were relatively low and the lining layer was thin. Thus, the transgenic mice that over-expressed CA1 showed more AS symptoms than RA symptoms following treatment with collagen.

Joint tissue destruction is a hallmark of inflammatory joint diseases, such as RA and AS. Despite considerably different pathologies, there are certain similarities among the diseases. In particular, inflammation and bone and cartilage destruction are the driving force for the structural changes in the diseased joints. We recently reported that the gene encoding CA1 is susceptible to AS and RA [10]. Thus, it is not surprised that we observed more AS symptom in CA1-Tg following collagen treatment. CIA is generally used to investigate mechanism of RA. Gillet et al. studied 40 inbred female Wistar Furth rats. At 2 weeks after immunization with native human type-II collagen, the rats showed polyarthritis that progressed to ankylosis associated with ossifying enthesopathy and periosteal new bone formation. Inflammatory nodules of the tail appeared after 2 months, with radiological and histopathological aspects of multistage spondylodiscitis [18]. Their finding supports the fact that collagen-induced arthritis may be a relevant model of peripheral and axial ossifying enthesopathy.

In the present study, we did not investigate the site of genome incorporation of CA1 in the transgenic mice. We prepared a series of controls including collagen-injected PADI4-Tg, BSA-injected CA1-Tg and collagen-injected wild-type. These controls showed low joint inflammation and no joint inflammation in their joint tissues. The controls also showed normal expression of CA1 in various organs of the mice. On the other hand, CA1-Tg showed significant joint inflammation following injection of collagen II. These results suggest that the CMV promoter and neighboring genes did not put an effect on the CA1 expression in CA1-Tg and did not take influence on the result of the study.

Our study had demonstrated that CA1 stimulates calcium salt precipitation and cell calcification which could explain the higher incidence of the disease in CA1 transgenic mice which can explain the joint tissue destruction [5]. We had also reported that CA1 encoding gene has a strong association with AS [10]. The present study detected high expression of CA1 in spleen, thymus and synovial tissues. But it is not sure if CA1 has any effect on adaptive immune response which could also explain the higher incidence of the disease in CA1 transgenic mice. Recently, Yamanishi H, et al. treated SCID mice with CA1-pulsed regulatory dendritic cells. The treated mice had higher mRNA expression of IL-10 and TGF-β1 and lower IL-17 mRNA expression [19].

## Conclusion

This study investigated the pathogenic role of CA1 using a transgenic animal model. Transgenic mice that over-expressed CA1 show an enhanced susceptibility to CIA. Histochemistry and X-ray analysis also demonstrated similar joint destruction to that found in AS. This finding may provide additional understanding of the pathogenic mechanism underlying the rheumatic disease.

## Abbreviations

CA1: Carbonic anhydrase I; CIA: Collagen induced arthritis; CA1-Tg: Transgenic mice with an over-expression of CA1; PADI4-Tg: Transgenic mice over-expressing PADI4; BSA: Bovine serum albumin; AS: Ankylosing spondylitis; OA: Osteoarthritis; cII: Collagen type II.

## Competing interests

The authors declare that they have no competing interests.

## Authors’ contributions

XC had full access to all of the data from this study and takes responsibility for the integrity of the data and the accuracy of the data analyses. YZ, LW and HX performed animal experiment. YZ, LW and WZ performed western blots, immunohistochemistry and real time PCR. All authors have read and approved the final manuscript for publication.

## Pre-publication history

The pre-publication history for this paper can be accessed here:

http://www.biomedcentral.com/1471-2474/13/256/prepub
